# Targeting Cancer Stem Cells and Hedgehog Pathway: Enhancing Cisplatin Efficacy in Ovarian Cancer With Metformin

**DOI:** 10.1111/jcmm.70508

**Published:** 2025-05-19

**Authors:** Emad Jafarzadeh, Vahideh Montazeri, Shima Aliebrahimi, Ahmad Habibian Sezavar, Mohammad H. Ghahremani, Seyed Nasser Ostad

**Affiliations:** ^1^ Department of Pharmacology and Toxicology Pharmacy, Tehran University of Medical Sciences Tehran Iran; ^2^ Artificial Intelligence in Medical Sciences Research Center Smart University of Medical Sciences Tehran Iran; ^3^ Toxicology and Poisoning Research Centre, Department of Toxicology and Pharmacology Pharmacy, Tehran University of Medical Sciences Tehran Iran

**Keywords:** autophagy, cancer stem cells, cisplatin, hedgehog signalling pathway, metformin, ovarian cancer

## Abstract

Ovarian cancer (OC) remains a leading cause of gynaecological cancer deaths due to late diagnosis and the emergence of resistance to platinum‐based chemotherapy, like cisplatin (Cis). Here, we investigated the potential of metformin (Met), a drug commonly used for type 2 diabetes, to overcome Cis resistance in OC. Our findings revealed a synergistic effect of Met with Cis in inhibiting cell viability, proliferation and colony/sphere formation capacity in both cisplatin‐sensitive (A2780) and ‐resistant (A2780/CDDP) ovarian cancer cell lines. This synergistic action triggered apoptosis through DNA damage, S‐phase cell cycle arrest and modulation of autophagy. Met also significantly decreased the expression of pluripotency transcription factors (Oct‐4, Sox2 and Nanog), indicating its potential to target cancer stem cells (CSCs). Furthermore, the combination therapy downregulated multidrug resistance protein 1 (MDR1) and excision repair cross‐complementation group 1 (ERCC1) expression, thereby sensitising resistant cells to Cis‐induced cytotoxicity. Additionally, the combination treatment suppressed the Hedgehog (Hh) signalling pathway, which is an important factor in inhibiting CSCs. Our study highlights the potential of the Met signalling pathway to synergise with Cis, overcoming therapeutic resistance in OC by targeting diverse cellular processes, including CSCs, and warrants further investigation in preclinical models.

## 1 Introduction

Ovarian cancer (OC) is a highly lethal gynaecological malignancy with a poor prognosis, particularly in advanced stages. Despite current treatments, including cytoreductive surgery and platinum‐based chemotherapy, the 5‐year survival rate has not significantly improved in recent decades. Research highlights the role of cancer stem cells (CSCs) in chemotherapy resistance and therapy failure [1].aberrant activation of the hedgehog (Hh) signalling pathway is implicated in OC. Normally inactive without ligands, Hh signalling is initiated by ligand binding to Patched (PTCH), relieving its inhibition of Smoothened (SMO) and triggering GLI‐mediated transcription, promoting tumour progression. Targeting this pathway offers a promising therapeutic strategy [2].

Cisplatin(Cis), a widely used chemotherapy agent, induces DNA damage but often encounters resistance. Combining Cis with Metformin (Met), an antidiabetic drug with anticancer properties, has shown potential in overcoming resistance, suppressing CSCs and enhancing apoptosis. Met's ability to target the HH pathway and reduce Cis resistance highlights its therapeutic synergy in OC treatment [3, 4, 5].

This study evaluates the combined effects of Met and Cis on both Cis‐sensitive and Cis‐resistant A2780 OC cell lines, hypothesising a synergistic impact that may suppress tumour growth and mitigate drug resistance at reduced Cis doses.

## 2 Materials and Methods

### 2.1 Cell Viability Assay

A2780 and A2780/CDDP cells (Cell Bank of Pasteur Institute of Iran) were seeded in 96‐well plates at a density of 7 × 103 cells/well and cultured for 24 h. After that, various concentrations of Cis ranging from 0.43 to 56 μM and Met ranging from 0.39 to 50 mM were prepared in RPMI‐1640 medium and added to the wells. Following a 48‐h incubation, the MTT assay was conducted. Lastly, the half‐maximal inhibitory concentration (IC50) of different treatments was calculated using GraphPad Prism 8 software.

### 2.2 Combined Drug Effect Analysis

The drug interaction between Met and Cis was analysed using Compusyn software based on the experimental method of Chou and Talalay, as well as Combenefit software [6]. The combination of Met and Cis at concentrations of 1/4, 1/2, 1 and 2‐fold the IC_50_ value of stand‐alone treatment with each drug was examined. The drug interactions were evaluated using the combination index (CI), where a CI value equal to 1 indicates an additive effect. A CI value > 1 signifies an antagonistic interaction, while a CI value < 1 reflects a synergistic interaction between the two compounds.

### 2.3 Apoptosis Analysis and Cell Cycle Analysis

These methods were performed as previously described [7].

### 2.4 Sphere and Colony Forming Assay

These assays were conducted as previously described [8, 9].

### 2.5 Real‐Time PCR Analysis

Gene expression levels of pluripotency factors (Nanog, Sox2, Oct‐4), Hh pathway markers (Gli, PTCH, SMO), drug resistance markers (ERCC1, MDR1) and housekeeping genes (GAPDH, β‐actin) were analysed in A2780 and A2780/CDDP cells via real‐time PCR (see File S1). RNA was extracted using Trizol, reverse transcribed into cDNA and amplified using the StepOne real‐time PCR system with SYBR Premix Ex Taq II. GAPDH and β‐actin were initially used as internal controls, but due to treatment effects on β‐actin, GAPDH was used exclusively for normalisation using the 2^−ΔΔCt method.

### 2.6 Evaluation of Autophagy

The presence and distribution of LC3‐II, a key marker for autophagy, were analysed in A2780 and A2780/CDDP cells using an indirect intracellular labelling technique. Cells treated with Cis, Met, and their combination were fixed with paraformaldehyde, permeabilised, and blocked with BSA. They were then incubated with an LC3‐II primary antibody (1:1000 dilution) followed by a secondary Alexa Fluor 488‐conjugated antibody. Nuclei were stained with DAPI, and fluorescence microscopy was used for analysis.

### 2.7 Statistical Analysis

Data are expressed as mean ± SEM and analysed using one‐way and two‐way ANOVA in GraphPad Prism 8, followed by a Student–Newman–Keuls post hoc test for group comparisons. Experiments were repeated at least three times, with statistical significance defined as *p* < 0.05.

## 3 Results

### 3.1 Effect of Combination Therapy on Cell Viability

**FIGURE 1:**
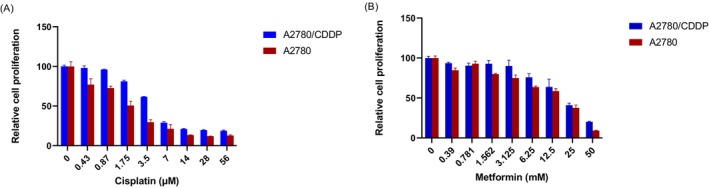
Cytotoxic effects of (A) Cisplatin (0.43–56 μM) and (B) Metformin (0.39—50 mM) in A2780 and A2780/CDDP cells for 48 h.

Figure 1 demonstrates the dose‐dependent growth inhibition of A2780 and A2780/CDDP cells upon treatment with Cis. The calculated IC_50_ values for Cis, after 48 h of treatment, were found to be 1.8 and 4.5 μM for A2780 and A2780/CDDP cells, respectively. When exposed to Cis concentrations ranging from 0.43 to 56 μM, A2780 cells exhibited growth inhibition at all concentrations, whereas A2780/CDDP cells experienced growth inhibition following treatment with concentrations greater than or equal to 1.75 μM.

In the case of Met, the results demonstrated an increase in the inhibitory effects of Met on cell growth with increasing concentrations. Figure 1B illustrates the IC_50_ values of Met in A2780 and A2780/CDDP cells, which were determined to be 19 and 21.5 mmol/L, respectively. Notably, the IC_50_ value of Met was similar in both cell lines, suggesting comparable sensitivity to Met. The IC50 values of Cis for A2780 and A2780/CDDP cells were 1.8 ± 0.7 and 4.5 ± 1.9, respectively. For Met, the IC50 values were 21.5 ± 3.4 for A2780 cells and 19 ± 2.1 for A2780/CDDP cells, respectively.

To determine the optimal concentration of Met for enhancing the efficacy of Cis, A2780 cells were simultaneously treated with different multiples (1/4, 1/2, 1 and 2‐fold) of the IC_50_ concentrations of Met (4.75, 8.5, 19, and 38 mM) and Cis (0.45, 0.9, 1.8 and 3.6 μM). Similarly, A2780/CDDP cells were treated with Met concentrations (5.375, 10.75, 21.5 and 43 mM) and Cis concentrations (1.125, 2.25, 4.5 and 9 μM).

### 3.2 Synergistic Effect of Met and Cis Combination in Ovarian Cancer

**FIGURE 2:**
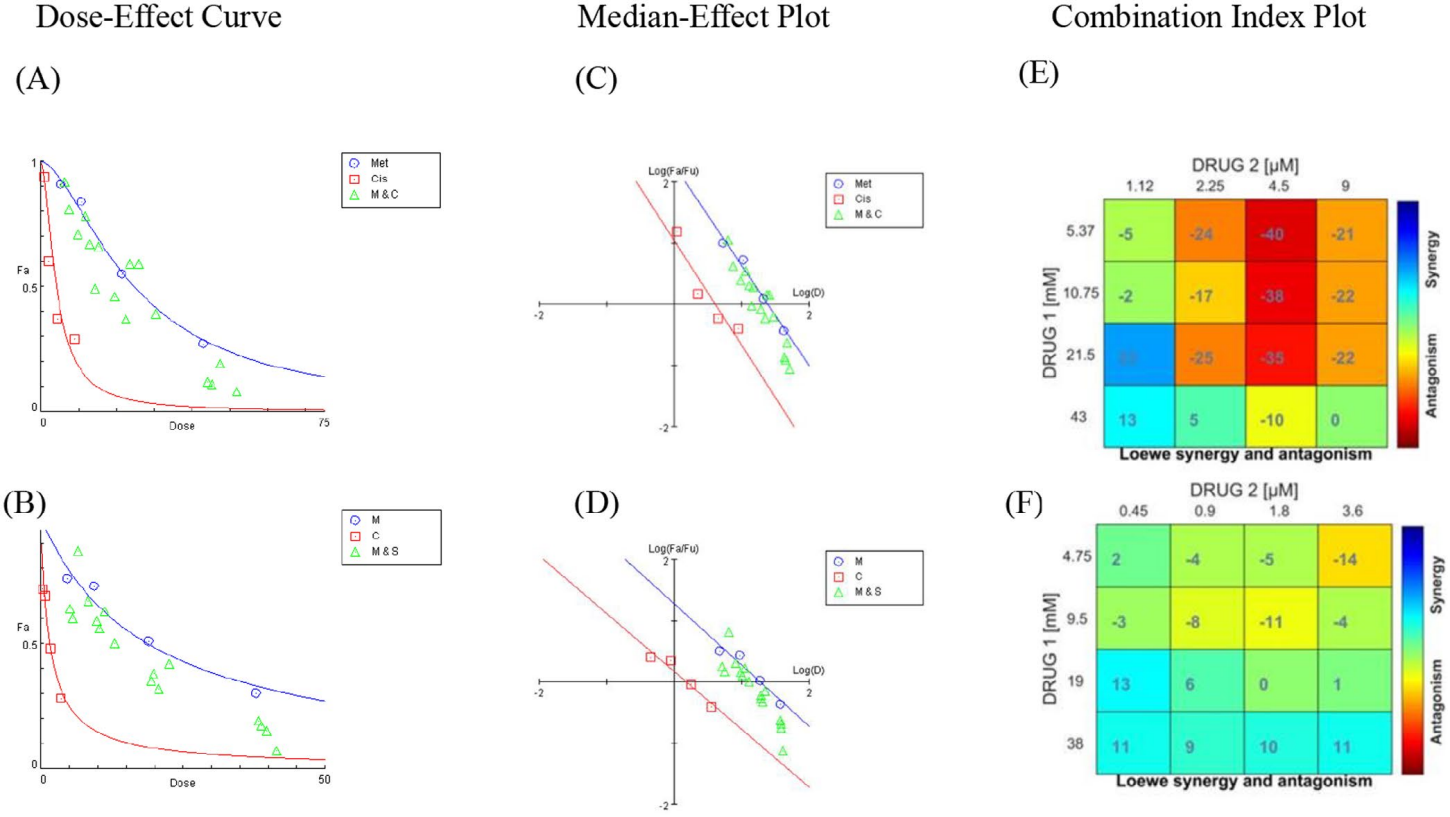
Dose‐response and combination therapy analysis for Met and Cis in A2780/CDDP and A2780 cells. (A, B) Dose‐response curves and median‐effect diagrams for Met and Cis, both as standalone treatments and in combination, were generated for A2780/CDDP (A) and A2780 (B) cells using the CompuSyn software. These diagrams were constructed utilizing the Chou and Talalay method. (C, D) Median‐effect diagrams of combination therapy for Met and Cis in A2780/CDDP and A2780 cells, respectively. (E, F) Isobologram analysis and synergy determination of combination therapy for Met and Cis in A2780/CDDP and A2780 cells, respectively, using the Combenefit software.

The combination of Met and Cis was analysed for synergistic or additive effects using a nonconstant ratio model with Compusyn and Combenfit software over a 48‐h incubation (Figure 2). Dose‐Effect Curves, Median‐Effect Plots and Combination Index (CI) analyses showed significant synergy. In A2780/CDDP cells, 21.5 mM Met combined with 1.12 μM Cis yielded a CI < 1, indicating synergy. Similarly, in A2780 cells, 19 mM Met with 0.45 μM Cis also showed a CI < 1, confirming synergistic interactions.

### 3.3 Synergistic Effects of Met and Cis on Apoptosis Induction and Cell Cycle Regulation

**FIGURE 3:**
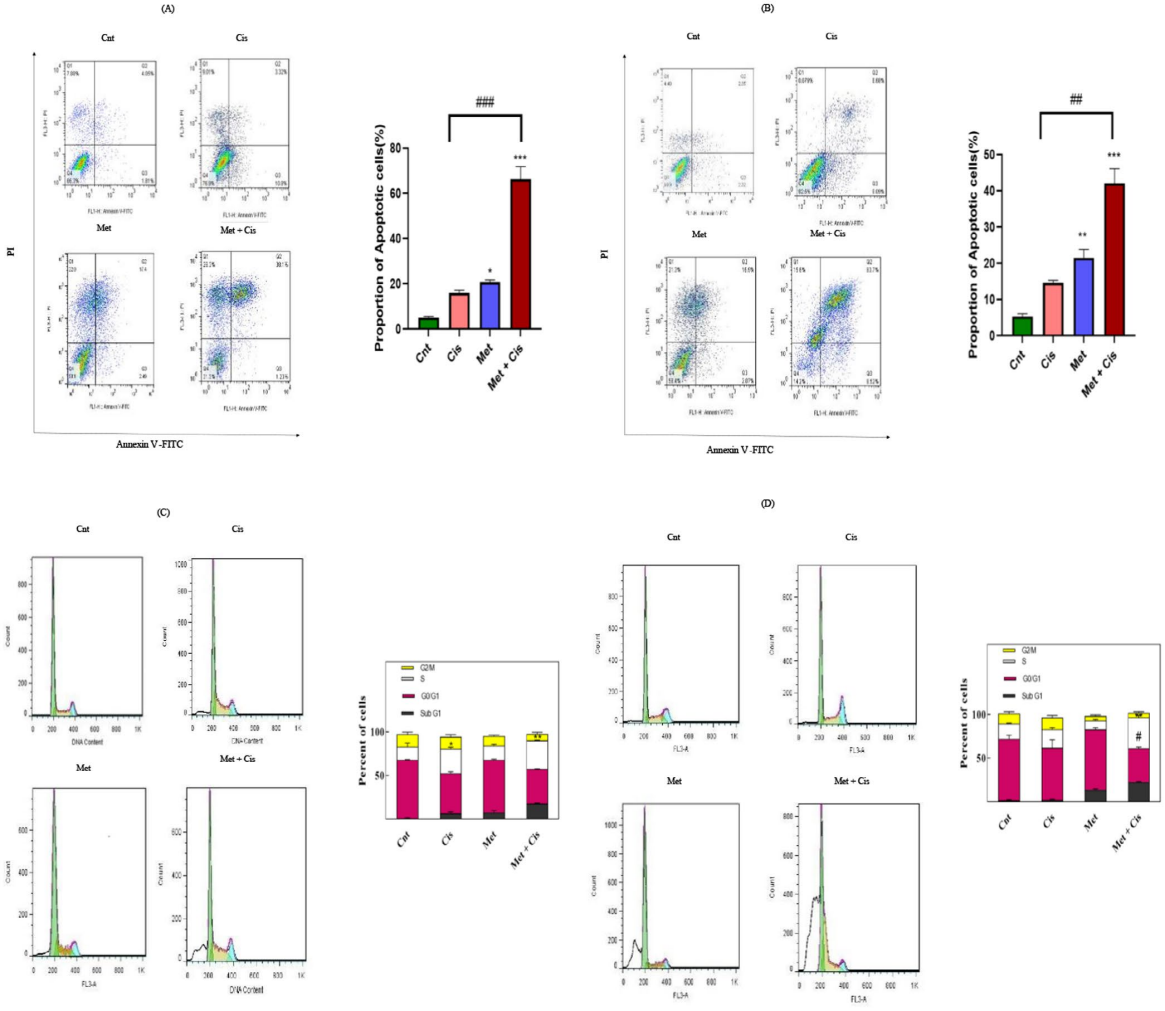
Effects of Met, Cis, and their combination on apoptosis and cell cycle distribution. (A) Apoptosis measurement in A2780/CDDP cells after treatment with Met, Cis, or their combination for 48 h, using flow cytometry with Annexin V and PI staining. (B) Apoptosis measurement in A2780 cells after treatment with Met, Cis, or their combination for 48 hours, using flow cytometry with Annexin V and PI staining. (C) Cell cycle distribution in A2780/CDDP cells after treatment with Met, Cis, or their combination for 48 h. (D) Cell cycle distribution in A2780 cells after treatment with Met, Cis, or their combination for 48 h. Data are presented as the mean ± standard deviation of three independent experiments. Significance was established at *p* ≥ 0.05. Two‐way ANOVA *p < 0.05, **p < 0.01, ***p < 0.001.

Annexin V‐PI staining was used to assess the effects of Met and Cis on apoptosis in A2780 and A2780/CDDP cells. Four groups (control, Met, Cis and Met+Cis) were analysed. The combination therapy significantly enhanced apoptosis compared to monotherapy. In A2780 cells, Cis (0.45 μM) + Met (19 mM) increased apoptosis sixfold (***p < 0.001). Similarly, in A2780/CDDP cells, Cis (1.12 μM) + Met (21.5 mM) boosted apoptosis threefold compared to Cis alone (**p < 0.01). As shown in Figure 3, Cis alone did not significantly raise apoptosis in either cell line.

The impact of Met and Cis combination treatment on the cell cycle was analysed in A2780 and A2780/CDDP cells using flow cytometry after 48 h of treatment. Treatment with 19 and 21.5 mM Met increased the G0/G1 phase cell population and reduced the G2/M phase population, though these changes were not statistically significant. Cis treatment (0.45 μM for A2780 and 1.12 μM for A2780/CDDP) caused over 25% of cells to arrest in the S phase (Figure 3).

The combination of Met and Cis further reduced the G2/M phase population while significantly increasing S phase accumulation in A2780 (44% ± 1.31%) and A2780/CDDP (33% ± 0.29%) cells, suggesting apoptosis induction (*p* < 0.01) (Figure 3C,D). Sub‐G1 phase analysis also showed a significant increase in apoptosis in the Met + Cis group compared to Cis alone in A2780/CDDP (*p* < 0.05) and A2780 (*p* < 0.01) cells, reinforcing the apoptotic effect of the combined treatment (Figure 3).

### 3.4 Targeting Stemness Properties With Met and Cis Combination Therapy

**FIGURE 4:**
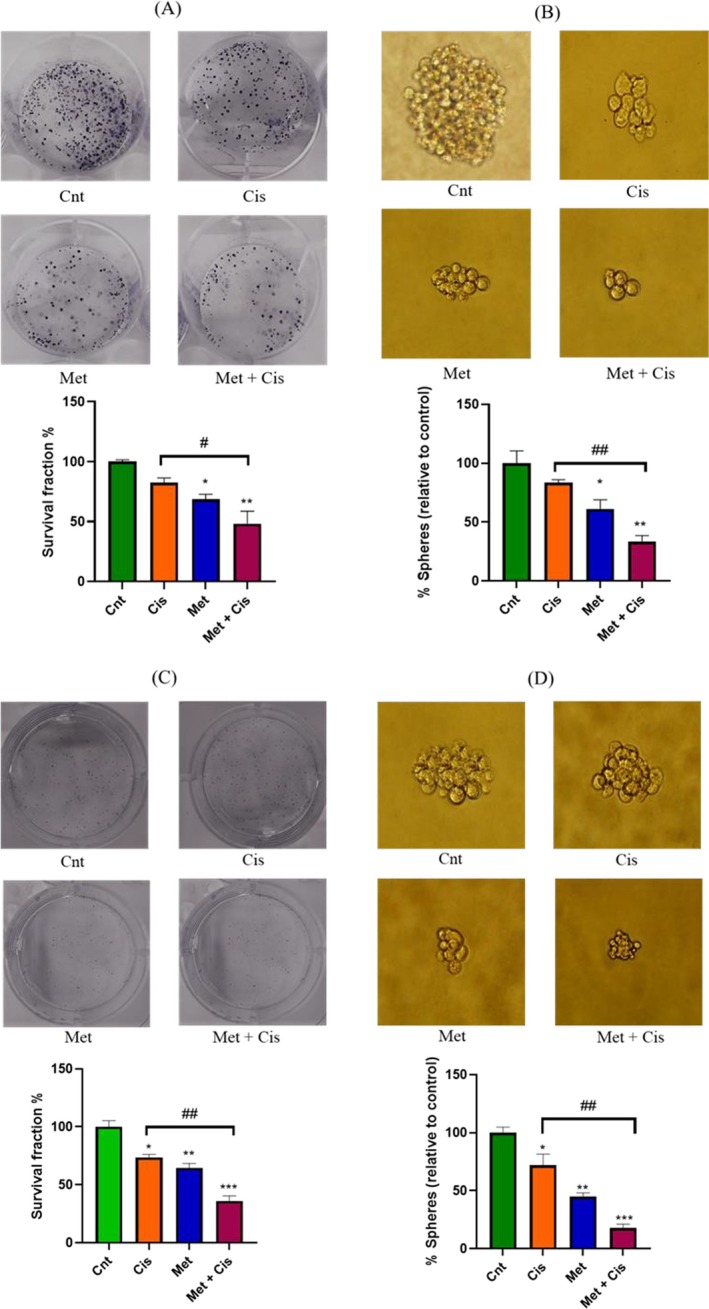
Downregulation of sphere and colony formation capacity by Met, Cis, and their combination in A2780/CDDP and A2780 cells. (A, B) A2780/CDDP cells were treated with the indicated concentrations of Met and Cis for 48 h in complete medium. Following treatment, (A) cells were plated for colony formation assay for 10 days, and (B) for sphere formation assay for 7 days (Magnification 20×). (C, D) A2780 cells were treated with the indicated concentrations of Met and Cis for 48 h in complete medium. (C) Following treatment, cells were plated for colony formation assay for 10 days, and (D) for sphere formation assay for 7 days (Magnification 20×).

The results highlight the impact of Met and Cis on self‐renewal capacity in A2780 and A2780/CDDP cells. The colony formation assay (Figure 4A,C) revealed that A2780 cells showed a greater reduction in colony formation than A2780/CDDP cells across all groups. Combined Met and Cis treatment significantly suppressed colony formation in A2780 (64.1 ± 5.1, **p < 0.01) and A2780/CDDP (51.7 ± 4.3, *p < 0.05) cells compared to Cis alone.

Similarly, the sphere formation assay (Figure 4B,D) demonstrated that the combined treatment reduced sphere formation efficiency by 82% (***p < 0.001) in A2780 cells and 66% (**p < 0.01) in A2780/CDDP cells, compared to reductions of 28% and 16%, respectively, with Cis alone. These results underscore the enhanced efficacy of the combined treatment in suppressing stemness‐related features.

### 3.5 Assessment of Autophagy Through the Utilisation of Indirect Immunofluorescence With LC3B Labelling

**FIGURE 5:**
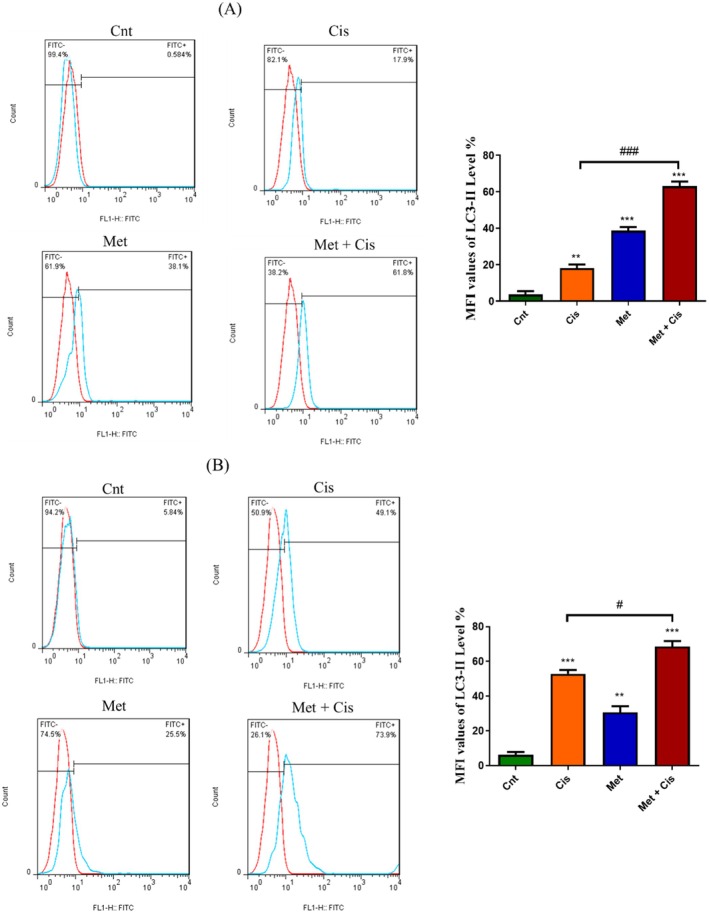
Histogram overlays depicting LC3II expression in isotype control, untreated cells, and cells treated with Met, Cis, and Met + Cis at 48 h. (A) MFI values of LC3II‐antibody for untreated and treated A2780/CDDP cells. (B) MFI values of LC3II‐antibody for untreated and treated A2780 cells. Data are presented as the mean ± standard deviation. Significance was determined using ANOVA, with *p < 0.05, **p < 0.01, ***p < 0.001.

Figure 5 illustrates the LC3‐II MFI response in A2780 and A2780/CDDP cells under various treatments. After 48 h, both Cis and Met significantly increased LC3‐II levels compared to the control group. In A2780/CDDP cells, the combination of Met and Cis produced a markedly higher LC3‐II signal than Cis alone (*p* < 0.001), indicating a synergistic effect. In A2780 cells, the combination showed a mild increase in LC3‐II levels compared to Cis alone (*p* < 0.05), with Cis alone having a similar impact to the combination. These results highlight that Met and Cis individually induce autophagy, but their combination enhances lysosome/autolysosome accumulation at the LC3‐II level, likely due to disrupted autophagic flux, linking the findings to the autophagy–lysosome pathway.

### 3.6 Synergistic Inhibition of Drug Resistance Genes and Pluripotency Factors by Met and Cis

**FIGURE 6:**
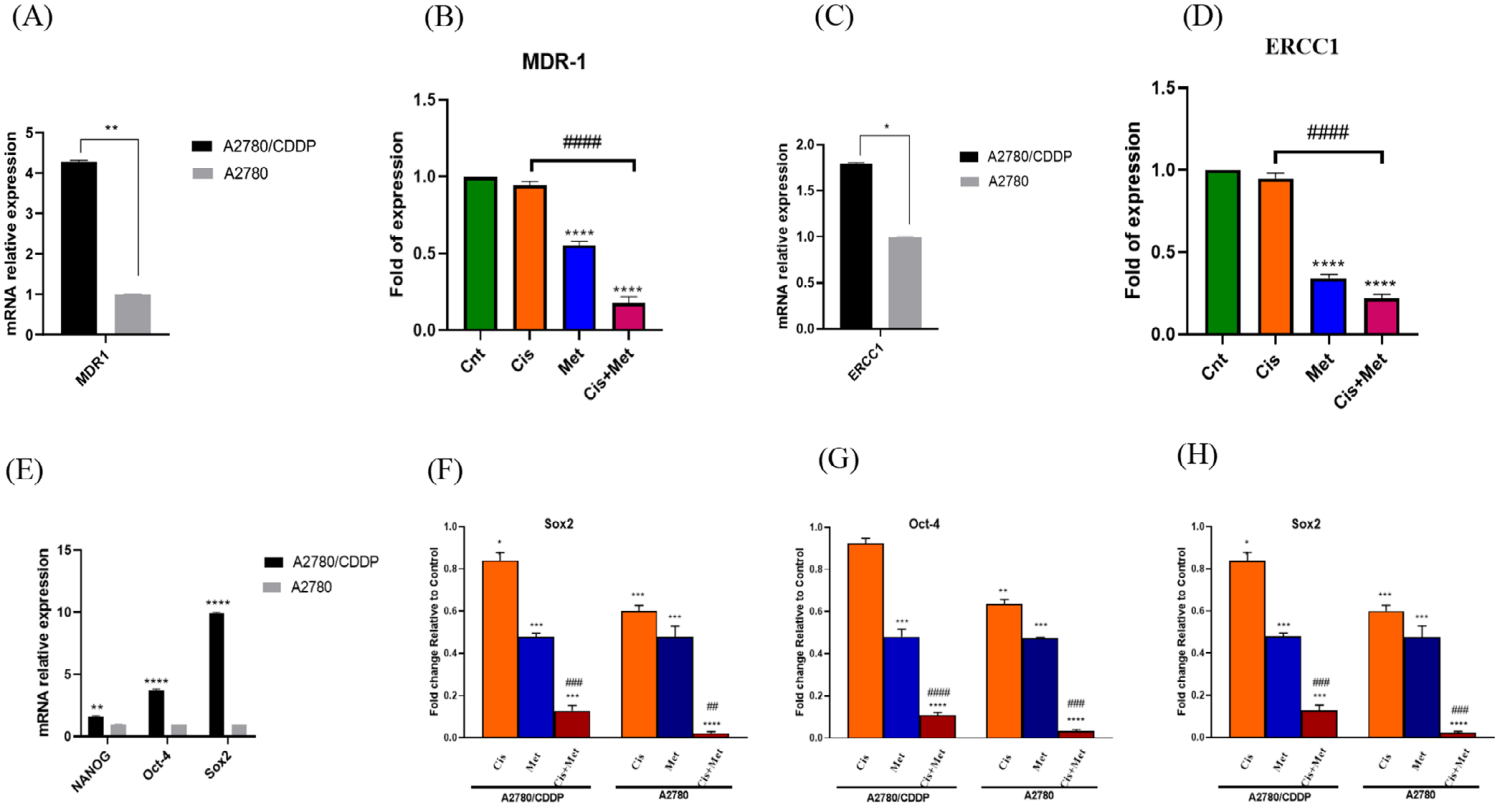
QPCR analysis of MDR1 and ERCC1 genes and pluripotency transcription factors. (A, C) Displays the differential expression levels of MDR1 and ERCC1 between A2780/CDDP and A2780 cells. (B, D) Shows the relative mRNA expression levels of MDR1 and ERCC1 in A2780/CDDP cells under various treatment conditions. (E) Illustrates the disparity in the expression of pluripotency transcription factors between A2780/CDDP and A2780 cells. (F, G and H) Graphical presentation of relative mRNA expression levels of pluripotency transcription factors (Nanog, Oct‐4, Sox2) in A2780/CDDP and A2780 cells, following exposure to different treatment groups.

Treatment with Met, Cis and their combination effectively suppressed drug resistance genes in A2780/CDDP cells. MDR1 mRNA expression showed a 4.3‐fold increase, and ERCC1 expression a 1.8‐fold increase in A2780/CDDP cells compared to A2780 cells, both of which were significantly inhibited by the treatments (Figure 6B–D). Similarly, the expression of pluripotency‐related genes Nanog, Oct‐4, and Sox2, elevated by 1.6‐fold, 3.7‐fold and 9.7‐fold respectively in A2780/CDDP cells, was markedly reduced with combination therapy compared to monotherapy (Figure 6E). These findings highlight the potential of Met and Cis to overcome drug resistance and induce apoptosis in resistant OC cells.

### 3.7 mRNA Expression of Hh Pathway Genes in Drug Resistance

**FIGURE 7:**
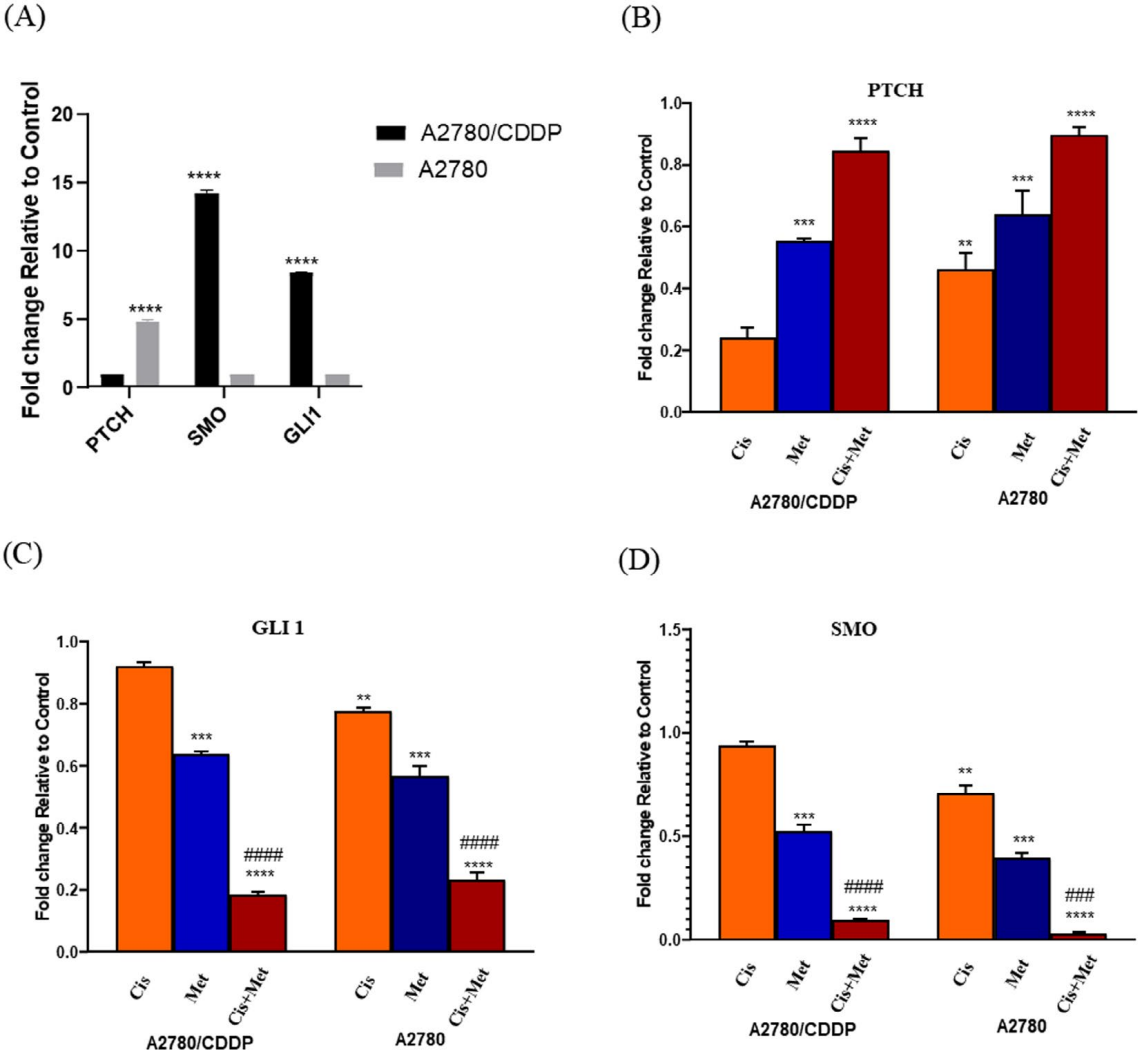
Displays the results of qPCR analysis conducted on the PTCH, SMO, and Gli1 genes in both A2780/CDDP and A2780 cells. (A) Illustrates the disparity in expression levels of Hedgehog signalling pathway between A2780/CDDP and A2780 cells. (B, C and D) The graphic presentation illustrates the relative expression levels of mRNA encoding Hedgehog signalling pathway in A2780/CDDP and A2780 cells, following exposure to various treatment groups.

The mRNA expression of Gli1, SMO and PTCH genes was evaluated to study the role of the Hh signalling pathway in drug resistance and CSC self‐renewal. In A2780/CDDP cells, Gli1 and SMO levels were significantly elevated by 14.25‐fold and 8.39‐fold, respectively, compared to A2780 cells, while PTCH expression was 4.87‐fold higher in A2780 cells (Figure 7A).after treatment with Met, Cis, and their combination, the Met + Cis group showed a synergistic effect, increasing PTCH expression and inhibiting Gli1 and SMO, leading to downstream suppression of target genes (Figure 7).

## 4 Discussion

Despite medical breakthroughs, during recent years, there has been a lack of substantial progress in reducing mortality rates associated with OC, as the 5‐year survival rate following diagnosis stands at approximately 47% [10]. OC is marked by its secretive nature and high proliferation and invasiveness. Despite aggressive surgery and chemotherapy, over 70% of patients face recurrence, with a median progression‐free survival of 12–18 months [11]. Therefore, there is a pressing need to develop novel and more efficacious drugs for this particular type of cancer. While single‐drug treatments may often lead to the development of drug resistance, on the other hand, combination therapies have demonstrated promising efficacy in inducing cancer cell death [12]. In our study, we found that the combined administration of Met and Cis sensitised Cis‐resistant OC cells, thereby enhancing treatment effectiveness. Met, an FDA‐approved drug for type 2 diabetes, has shown antitumour properties. Emerging evidence suggests that Met plays a significant role in adjuvant tumour treatment, augmenting the sensitivity of conventional chemotherapy and immunotherapy. Furthermore, combining Met with other anticancer drugs has been shown to enhance antitumour effects [13]. In our current investigation, we explored the application of Met in reversing Cis resistance in OC cells. Specifically, we utilised both Cis‐resistant (A2780/DDP) and wild‐type (A2780) human OC cell lines.

The concept of repurposing Met in the field of cancer has gained significant attention due to its favourable safety profile, epidemiological data, and promising outcomes observed in clinical trials for the treatment of several types of cancer, where treatment options are limited [14]. Our in vitro cytotoxicity assay demonstrated the profound inhibitory effects of Met on OC cell lines. Notably, Met exhibited similarly significant inhibition of cell viability in both A2780 and A2780/CDDP cell lines after 48 h of exposure (Figure 1), which was consistent with the results reported by dos Santos Guimarães et al. regarding Cis‐ and paclitaxel‐resistant OC cells [15].

Co‐administration of Met with anticancer agents shows significant synergy, overcoming chemoresistance in various cancers. At micromolar concentrations, Met synergistically enhances carboplatin cytotoxicity in OC cell lines, suggesting a promising therapeutic combination [16]. Also, a study by He et al. reports that a combination of Met and Cis can lower the administered doses of Cis and, as a result, increase the sensitivity of testicular germ cell tumours to Cis [17]. Similarly, in the present investigation, our findings indicated a pronounced enhancement in the inhibition of cell viability for A2780 and A2780/CDDP cells when Met was administered in conjunction with Cis, surpassing the outcomes observed with stand‐alone treatments of Met or Cis. Furthermore, our study revealed that Met significantly augmented the chemosensitivity of Cis‐resistant cells to Cis, resulting in substantial suppression of cell proliferation in both cell lines.

The administration of Met in conjunction with Cis for the treatment of OC cells, both sensitive and resistant to Cis, resulted in a marked increase in apoptosis, as assessed through Annexin V‐PI staining. This synergistic apoptotic effect aligned with findings in the existing scientific literature, stressing the potential efficacy of this combined treatment approach [3]. Particularly at the specified concentrations, the combination of Met and Cis demonstrated a statistically significant augmentation of apoptosis compared to the use of Cis alone, which was in accordance with prior investigations [18], suggesting a potential capacity to overcome resistance mechanisms associated with Cis therapy. A potential assortment of molecular mechanisms, including the impact on DNA synthesis and repair, coupled with Met‐induced metabolic disturbances, may contribute to the observed enhanced apoptotic response [19].

Mechanistically, Cis interferes with DNA synthesis and repair, resulting in cell cycle arrest, particularly in the S phase. Simultaneously, the impact of Met on energy metabolism and mTOR signalling pathways is evidenced by an increased proportion of cells in the G0/G1 phase [20]. Our cell cycle analysis showed that the combination resulted in S phaseell cycle arrest and simultaneously reduced the percentage of cells in the G2/M phases. As mentioned earlier, these results might stem from the induction of DNA damage by Cis, interfering with DNA synthesis, and the metabolic modifications facilitated by Met. The considerable shift towards the S phase following combined treatment implied a potential hindrance to DNA replication and repair processes. The notable rise in S phase cells signified an intensified apoptotic response, underscoring the potential of this combination in overcoming Cis resistance in OC cells. The same outcomes were reported in a study by Bi et al., where combination therapy with Cis and Met gave rise to a G0/G1 phase cell cycle arrest in gallbladder cancer cells by upregulation of P21, P27 and downregulation of CyclinD1.

It has been reported that Met can affect stemness properties as it reduces the expression levels of CD44, a well‐known stemness‐related marker, in cancer cells [21]. To assess the impact of Met alone or in combination with Cis on the stemness properties of A2780/CDDP and A2780 cells, we investigated the mRNA expression levels of pluripotent transcription factors, namely Nanog, Sox2, and Oct‐4. These transcription factors play a critical role in regulating pluripotency‐associated characteristics. Within A2780/CDDP and A2780 cells, Oct‐4 functions as a key regulator of pluripotency and has been shown to induce apoptosis in malignancies via the Oct‐4/Tcl1/Akt1 pathway [22]. Additionally, high Nanog expression has been linked to various CSC properties, including EMT, cell proliferation, migration, and resistance to chemotherapy [23]. Similarly, overexpression of Sox2 has been linked to increased cancer invasion, resistance to radiation, and chemotherapy, ultimately leading to a poor prognosis [24]. The results of the present study showed that inhibiting the expression of Oct‐4, Nanog and Sox2, following the combined treatment of synergistic doses of Met and Cis in both cell lines, especially resistant cells, due to high levels of these markers in them by nature, led to a decrease in self‐renewal capacity, which could lead to the induction of apoptosis as discussed earlier. To confirm the mRNA expression analysis, colony‐ and sphere‐formation assays were to be conducted. The co‐treatment significantly reduced colony and sphere numbers in both cell lines compared to Cis alone, aligning with Sung‐Hee Kim et al.'s findings, where Met with 5‐Fu significantly diminished colony and sphere formation [25].

Previous reports have indicated that Met inhibits autophagy in various cell lines [26]. To investigate autophagy following treatment with the combination, the prominent autophagy marker, LC3‐II, was quantified through flow cytometry. The results distinctly demonstrated that the application of Met and Cis, at doses that induced synergism, induced similar autophagy responses in both Cis‐resistant and Cis‐sensitive OC cell lines. Also, our data unequivocally expressed that autophagy played a regulatory role in mediating Cis resistance in human OC cells. Additionally, the synergistic effect not only induced apoptosis but also sensitised resistant OC cells. Furthermore, MDR1 appears to serve as a ubiquitous cellular response marker to chemotherapy across diverse cancer types. The significantly higher MDR1 expression levels in the Cis‐resistant cells were notably attenuated by the administered doses, particularly through the synergistic combination [27]. This observation implied a promising therapeutic synergism between Met and Cis in counteracting drug resistance in OC. In a similar manner, other studies suggested that Met might increase the incidence of autophagy in OC cells [28].

Analysing ERCC1 gene expression in both sensitive and resistant cells following treatment with Met, Cis, and their synergistic combinations could provide valuable insights. In sensitive cells, Cis triggers DNA damage, leading to increased ERCC1 expression as a repair mechanism. Met may further suppress ERCC1 levels, potentially amplifying the DNA damage response. Our findings revealed that synergistic Met and Cis doses entirely inhibited ERCC1 expression in sensitive cells. Interestingly, resistant cells, characterised by inherently higher ERCC1 levels compared to sensitive cells, still responded to the Met and Cis combination compared to Cis alone. This suggests that the combination therapy can significantly reduce ERCC1, potentially overcoming established resistance and sensitising resistant cells to Cis‐induced damage. These observations align with a study by Li et al., where the combination of Met and Cis demonstrated a reduced ERCC1 expression level compared to monotherapy with either drug [29].

The Figure 7 shows that the mRNA expression levels of Gli1 and SMO, two important genes in the Hh pathway, were significantly higher in the resistant cells compared to the cisplatin‐sensitive cells. This suggests that the Hh signalling pathway may be activated in cisplatin‐resistant CSCs.

Treatment with the combination of Cis and Met resulted in a significant upregulation of the PTCH gene, which is an inhibitor of the SMO gene. This suggests that the combination therapy may be working by inhibiting the Hh signalling pathway. The Hh signalling pathway plays a pivotal role in governing stem cell maintenance, self‐renewal processes and intricate interactions within the tumour microenvironment. Besides, this signalling pathway reportedly increases drug resistance via MDR1 in cancer [30]. These multifaceted roles underscore the significant contribution of the Hh pathway to essential cellular mechanisms relevant to cancer development. Interestingly, it has been reported that Met can manifest anticancer effects via inhibition of the Hh signalling pathway in breast cancer [31]. Elevated levels of Gli1, SMO, and MDR1 in chemotherapy‐resistant cell lines suggest activation of the Hh signalling pathway in OC. Our findings indicate that the Cis and Met combination could be a promising approach to overcoming Cis resistance, though further research is required to confirm these results and elucidate the underlying mechanisms.

## 5 Conclusion

Our findings highlight the synergistic potential of Met and Cis in overcoming Cis resistance through multifaceted mechanisms. The combination significantly enhances therapeutic efficacy by reducing cell viability, suppressing proliferation, and increasing apoptosis linked to DNA damage, cell cycle disruption, and autophagy modulation. Met disrupts CSCs self‐renewal, as evidenced by reduced sphere and colony formation and downregulation of pluripotent transcription factors. Effects on MDR1 and ERCC1 expression further sensitise resistant cells to Cis‐induced damage. The involvement of Hh signalling underscores its role in therapeutic resistance and presents a potential target for intervention. While promising, in vivo studies and clinical trials are essential to validate these findings and advance OC treatment strategies.

## Author Contributions

**Emad Jafarzadeh:** conceptualization (equal), data curation (equal), formal analysis (equal), investigation (equal), methodology (equal), project administration (equal), software (equal), validation (equal), writing – original draft (equal), writing – review and editing (equal). **Vahideh Montazeri:** conceptualization (equal), data curation (equal), methodology (equal), resources (equal), supervision (equal). **Shima Aliebrahimi:** data curation (equal), formal analysis (equal), investigation (equal), methodology (equal), software (equal), supervision (equal), visualization (equal), writing – review and editing (equal). **Ahmad Habibian Sezavar:** data curation (equal), formal analysis (equal), software (equal), validation (equal). **Mohammad H. Ghahremani:** conceptualization (equal), formal analysis (equal), investigation (equal), project administration (equal), supervision (equal), validation (equal), visualization (equal). **Seyed Nasser Ostad:** conceptualization (equal), formal analysis (equal), funding acquisition (equal), investigation (equal), methodology (equal), project administration (equal), resources (equal), supervision (equal), visualization (equal), writing – review and editing (equal).

## Ethics Statement

This study with the code IR.TUMS.TIPS.REC.1399.153 was registered at the Ethics Committee of the National Institute for Medical Research Development.

## Compliance With Ethical Standards

This article does not contain descriptions of studies performed by the authors with the participation of humans or using animals as objects.

## Conflicts of Interest

The authors declare no conflicts of interest.

## Supporting information


Appendix S1.

## Data Availability

The data that support the findings of this study are available from the corresponding author upon reasonable request.
