# Supplementary material for: Targeting Cancer Stem Cells and Hedgehog Pathway: Enhancing Cisplatin Efficacy in Ovarian Cancer With Metformin
Source: J Cell Mol Med. 2025 May 19;29(10):e70508. doi: 10.1111/jcmm.70508 (PMC12087300; doi:10.1111/jcmm.70508)
Supplement: Supplementary file 1 — Appendix S1. [file JCMM-29-e70508-s001.docx]

Supplementary File 1. Primer sequences used for real-time PCR

| Gene | Primer (Oligo Sequence 5'--> 3') | |
| --- | --- | --- |
| GLI1 | Forward | AGCCTGAATCTGTGTATGAAACTG |
|  | Reverse | GCTCGCTGTTGATGTGGTG |
| SMO | Forward | GTGGATGGGGACTCTGTGAG |
|  | Reverse | GTGGTTGCTCTTGATGGAGAAC |
| PTCH | Forward | GCACTACTTCAGAGACTGGCTTCA |
|  | Reverse | CCAGGAGTTTGTAGGCAAGGACT |
| ABCB1(MDR1) | Forward | CCCATCATTGCAATAGCAGG |
|  | Reverse | TGTTCAAACTTCTGCTCCTGA |
| ERCC1 | Forward | TTTGGCGACGTAATTCCCGAC |
|  | Reverse | CCTGCTGGGGATCTTTCACA |
| Sox2 | Forward | CATGACCAGCTCGCAGACCTA |
|  | Reverse | GGACTTGACCACCGAACCCA |
| Oct-4 | Forward | TTGCTGCAGAAGTGGGTGGA |
|  | Reverse | ATCTGCAGTGTGGGTTTCGG |
| Nanog | Forward | GCAGAGAAGAGTGTCGCAAAA |
|  | Reverse | CAGCTGGGTGGAAGAGAACA |
| GAPDH | Forward | GAAGGTGAAGGTCGGAGTCAAC |
|  | Reverse | CAGAGTTAAAAGCAGCCCTGGT |
| Β-actin | Forward | CTCACCATGGATGATGATATCGC |
|  | Reverse | CACATAGGAATCCTTCTGACCCA |
